# Aflibercept versus Faricimab in the Treatment of Neovascular Age-Related Macular Degeneration and Diabetic Macular Edema: A Review

**DOI:** 10.3390/ijms23169424

**Published:** 2022-08-20

**Authors:** Sławomir Liberski, Małgorzata Wichrowska, Jarosław Kocięcki

**Affiliations:** 1 Department of Ophthalmology, Poznan University of Medical Sciences, ul. Augustyna Szamarzewskiego 84, 61-848 Poznan, Poland; 2 Doctoral School, Poznan University of Medical Sciences, ul. Bukowska 70, 60-812 Poznan, Poland

**Keywords:** aflibercept, faricimab, anti-VEGF, angiopoietin-2, DME, diabetic macular edema, nAMD, neovascular age-related macular degeneration, Ang/Tie-2 pathway

## Abstract

Diabetic macular edema (DME) and neovascular age-related macular degeneration (nAMD) are common retinal vascular diseases responsible for most blindness in the working-age and older population in developed countries. Currently, anti-VEGF agents that block VEGF family ligands, including ranibizumab, bevacizumab (off-label use), brolucizumab, and aflibercept, are the first-line treatment for nAMD and DME. However, due to the complex pathophysiological background of nAMD and DME, non-response, resistance during anti-VEGF therapy, and relapses of the disease are still observed. Moreover, frequent injections are a psychological and economic burden for patients, leading to inadequate adhesion to therapy and a higher risk of complications. Therefore, therapeutic methods are strongly needed to develop and improve, allowing for more satisfactory disease management and lower treatment burden. Currently, the Ang/Tie-2 pathway is a promising therapeutic target for retinal vascular diseases. Faricimab is the first bispecific monoclonal antibody for intravitreal use that can neutralize VEGF and Ang-2. Due to the prolonged activity, faricimab allows extending the interval between successive injections up to three or four months in nAMD and DME patients, which can be a significant benefit for patients and an alternative to implanted drug delivery systems.

## 1. Introduction

In developed countries, diabetic macular edema (DME) and age-related macular degeneration (AMD) are common retinal vascular diseases responsible for the majority of cases of blindness and visual disability in the working-age and elderly population, respectively [1,2]. The common pathophysiological features of these conditions are abnormalities in the microcirculation of the retina, resulting in the growth of abnormal vessels characterized by impaired wall integrity. In neovascular AMD (nAMD), responsible for 90% of blindness among all AMD patients, pathological choroidal vessels grow beneath the macula, known as macular neovascularization (MNV), and is manifested clinically as fluid leakage and hemorrhage, which in effect leads to fibrous scarring. On the other hand, the dominant pathophysiological component in DME is increased vascular permeability, resulting in fluid leakage and the thickening of the retina in the macula, along with the possible formation of new, abnormal vessels derived from the retina [3]. Vascular endothelial growth factor (VEGF) is known to be the major mediator responsible for the development of pathophysiological changes in both DME and wet age-related macular degeneration (nAMD) [4]. Thus, the development of intravitreal anti-VEGF therapy in the last decade has revolutionized the treatment options for retinal vascular diseases, allowing the drug to be delivered directly into the eyeball, thus minimizing systemic side effects resulting from the targeted blocking of VEGF [5,6]. Due to high efficacy and safety, anti-VEGF monotherapy became the first-line treatment for nAMD and DME. It replaced the previously used clinical interventions, including laser photocoagulation and photodynamic therapy, which were less effective and associated with a higher recurrence rate in the presence of neovascular lesions and the incidence of complications [1,7].

To date, intravitreal anti-angiogenic agents used in clinical practice are based on limited activity against factors belonging to the VEGF family, including the inhibition of VEGF-A activity for ranibizumab, bevacizumab, and brolucizumab, as well as aflibercept, which has a broader spectrum of action and the ability to neutralize, in addition to VEGF-A, other VEGF family ligands—VEGF-B, placental growth factor-1 (PlGF-1), and placental growth factor-2 (PlGF-2) [8,9]. However, due to the complex pathophysiological background of nAMD and DME involving multiple signaling pathways, non-response and resistance during anti-VEGF therapy, as well as relapses of the disease provoking loss of initial visual acuity improvement, secondary to the exacerbation of neovascular lesions and vascular leakage, are still observed during anti-VEGF therapy [Sharma; Ng]. According to estimates, non-responders to treatment with anti-VEGF agents may reach 8.1–15% and 30–72% of nAMD and DME patients, respectively [8,10]. In the case of intravitreal aflibercept therapy, the prolonged half-life of the drug in the vitreous achieved due to the modification of the molecular structure allowed for extending the interval between injections to eight weeks both in nAMD and DME after the initial loading phase [10,11]. However, frequent injections are a significant psychological and economic burden for patients, with a lower rate of adequate adhesion to therapy and a higher risk of complications [12]. Therefore, despite the substantial progress in treating retinal vascular diseases, there is still a need to search for new therapeutic methods that provide adequate disease management to improve patients’ quality of life by reducing the treatment burden [13].

Recent studies have identified the angiopoietin/tyrosine kinase with immunoglobulin and epidermal growth factor homology domain receptor-2 (Ang/Tie-2) pathway as one of the most promising therapeutic targets for retinal vascular diseases [14]. One of the strategies focused on blocking angiopoietin-2 (Ang-2) activity that allows for the inhibition and further normalization of pathological changes in the retinal vasculature [2,15]. Faricimab is the first bispecific monoclonal antibody for intravitreal use and can neutralize both VEGF and Ang-2 [2]. Due to the prolonged activity after intravitreal administration, faricimab, following the results of phase III clinical trials, allows extending the interval between successive injections up to three or four months in nearly 80% and 70% of nAMD and DME patients, respectively [16,17]. Developing anti-VEGF therapy by introducing agents with prolonged activity into clinical use and allowing less frequent injections may be a competitive alternative to new therapies involving extensive surgery requiring implanted drug release ports [18]. Thus, reducing the treatment burden due to less frequent injections may improve patients’ quality of life and provide better adhesion to the therapy. Previous reviews separately summarized the use of aflibercept and faricimab in the therapy of nAMD and DME. In this paper, we summarize the current state of knowledge concerning using both aflibercept and faricimab in treating DME and nAMD, taking into account the published results of the phase III clinical trials in which these drugs were directly compared.

## 2. nAMD and DME as Global Health Care Problems

In developed countries, AMD is the most common cause of blindness among people aged 60 years or over [19]. According to epidemiological data, the incidence of AMD increases with age, and in the age group of 65–74 years, it reaches 11%, while in the population over 74 years, it affects up to 28% of individuals [4]. AMD is responsible for 7–8% of all blindness worldwide, placing it in third place after cataracts and glaucoma [4,19,20]. nAMD, also known as wet AMD, is much more likely to lead to irreversible loss of central vision than the dry form (dry AMD). Although nAMD occurs in 10–20% of all AMD patients, this type, due to the rapidly progressive destructive changes associated with the appearance of abnormal vessels under the macula, accounts for the irreversible loss of central vision in 90% of all individuals affected by this disease [4,21]. The predictions for the prevalence of nAMD in the world’s population are highly unfavorable, indicating a significant increase secondary to the aging population in the coming decades, especially in developed countries [1]. In 2020, the incidence of nAMD was 196 million, while by 2040, an increase of over 90 million new cases is expected [19].

Diabetes mellitus is one of the major health problems among the working-age population worldwide [22]. According to estimates, 382 million people worldwide have diabetes [23], and the number of individuals with diabetes is projected to reach 522 million in 2030, with a further projected expansion to 592 million in 2035 [23], and 642 million in 2040, which will be associated with the occurrence of diabetes in 1 in 10 adults worldwide [24]. Vascular damage in the course of diabetes mellitus is a well-known complication of diabetes and may occur in the form of macro- and microangiopathy [25]. Diabetic retinopathy (DR) is the most common form of diabetic microangiopathy and is defined as the appearance of pathological changes in the microcirculation of the retina in diabetic eyes. DR is the most common cause of vision loss among people of working age and, according to statistics presented by World Health Organization (WHO), is responsible for 4.8% of all blindness worldwide [15,23,26]. Two main types of DR, non-proliferative (NPDR) and proliferative (PDR), characterized by neovascular lesions are distinguished [26].

DME resulting from vascular leakage is the most common manifestation of DR leading to visual impairment in diabetic eyes and can occur at any stage of DR; however, it has been proven that DME is frequently found in patients with a more advanced form of DR [26,27,28,29]. DME is considered the leading cause of vision loss among working-age individuals in the developed world [13] and is estimated to affect 7.5% of diabetics, representing 21 million patients worldwide [30]. It has been shown that the incidence of DME rises with the duration of diabetes [31]. According to the results of the Wisconsin Epidemiologic Study of Diabetic Retinopathy, in a ten-year perspective, DME will occur in 20.1% of patients with type I diabetes and 25.4% and 13.9% of patients with insulin-dependent and insulin-independent type II diabetes, respectively [32]. In the case of type I diabetes, 29% of patients will suffer from DME within 25 years [32]. Thus, due to the predicted significant growth in the incidence of diabetes in the coming decades, the aging of the global population, as well as the expected increase in the life expectancy of diabetic patients associated with better care and more advanced treatments [23], the prognosis of the prevalence of DME in the following years is alarming [33].

Therefore, due to the increasing incidence of both nAMD and DME, which are associated with a marked impact on the health status of the global population, there is a strong need for the development and improvement of therapeutic methods that could allow for more satisfactory disease management and lower treatment burden, resulting in a better quality of patients’ life [33].

## 3. Pathogenesis of nAMD and DME

The pathogenesis of both DME and nAMD has been extensively studied in recent years. However, due to the complex background of both of these conditions, they are still not fully understood. Nevertheless, the leading hypothesis is based on the dominant destructive effect of VEGF overexpression and other pro-angiogenic factors secondary to local retinal ischemia [33].

### 3.1. nAMD

In the dry form of AMD, the first symptom of retinal abnormalities is drusen formation between the basal lamina of retinal pigment epithelium (RPE) and the collagen layer of the inner Bruch’s Membrane (BM) [20]. Drusen are focal deposits consisting of proteins, lipoproteins, and lipids [20] and can be observed on fundus examination or an optical coherence tomography (OCT) scan much earlier than visual impairment occurs [21]. Another crucial structural change in the retina in the early stage of AMD development is a thickening of BM [20]. This structure is the principal barrier to the penetration of new, pathological vessels and also plays an essential role in cellular transmission, tissue remodeling processes, and oxygen exchange between the retina and choriocapillaries. In addition, BM also plays a vital role in transporting nutrients and metabolites between the RPE/photoreceptor complex and the choroidal circulation [20,34]. Apart from the process of drusen formation, other factors leading to BM damage are the immune response secondary to the activation of the complement system and the increase in the expression of pro-inflammatory cytokines [21].

In wet AMD, a decrease in BM permeability leads to a reduction in retinal oxygenation and local hypoxia, which, together with an increase in proinflammatory cytokines, results in a secondary tissue response manifested by an increase in the expression of pro-angiogenic factors, especially VEGF [Flores]. VEGF is produced and released by several retina layers [20,21]. In addition to stimulating the growth of new vessels, VEGF enhances vascular permeability and promotes the proliferation and migration of vascular endothelial cells [21]. When local vascular homeostasis is disturbed by increased pro-angiogenic factors, the damaged BM structure is not an effective barrier against entering new, abnormal vessels from the choroid or the outer retina [20]. These pathological vessels have a weakened vascular wall structure that promotes the formation of hemorrhages and results in the leakage of extracellular fluid into the subretinal and intraretinal space [21]. These processes trigger an immune response, damaging the RPE layer and photoreceptors with subsequent atrophy, fibrosis, and macular scar formation, causing irreversible loss of central vision [21].

### 3.2. DME

Chronic hyperglycemia secondary to diabetes mellitus leads to the development of vascular system pathology in the body, both in the area of large vessels and the smaller vascular bed, and is responsible for the occurrence of complications classified as vascular macro- and microangiopathies, respectively [35]. DR is one of the most common microangiopathic complications in diabetics, and abnormalities secondary to pathological processes in retinal vascularization discovered during fundus examination are frequently the first symptoms of chronic systemic hyperglycemia [35]. DME can occur in all stages of diabetic retinopathy and is manifested as macular thickening secondary to the accumulation of intraretinal fluid (IRF) and subretinal fluid (SRF), the appearance of which is a result of the disruption of the blood-retinal barrier integrity and leakage of fluid from the retinal capillaries characterized by impaired endothelial integrity [26]. DME is believed to be driven by the parallel action of three destructive factors, including persistent hyperglycemia, the influence of pro-inflammatory factors, and neurodegenerative changes in the retina [26].

Chronic hyperglycemia is considered the primary determinant of pathological changes in the retinal microvasculature in DME [13]. The negative effect of chronically elevated glucose on body tissues occurs mainly through the increased activity of the polyol pathway, as well as through protein kinase C (PKC) and hexosamine biosynthetic pathways (HBP), as well as the accumulation of advanced glycation end products (AGEs), also known as glycotoxins. AGEs are a heterogeneous group of compounds forming during non-enzymatic glycations between carbonyl-reducing groups of sugars and free amino groups of nucleic acids, proteins, and lipids [36]. AGEs may adversely impact vascular tissue by influencing intracellular signaling pathways, resulting from interaction with cell surface receptors and the ability to interfere with the regulation of gene expression. Further, AGEs can increase the expression of pro-inflammatory cytokines and the local concentration of reactive oxygen species (ROS) [36]. Persistent hyperglycemia leads to disturbances in blood flow and, consequently, local ischemia and secondary hypoxia of the inner layers of the retina. Local tissue hypoxia results in the dilatation of the retinal vessels with a consequent increase in hydrostatic pressure in the venous vessels and capillaries, which leads to damage to their walls and subsequent occlusion [13,28]. In response to local oxygen deficiency, the hypoxia-inducible factor-1 (HIF-1) pathway is activated, inducing VEGF overexpression [26].

Additionally, it has been documented that hyperglycemia can trigger an increase in phospholipase A2 (PLA2) and secondary promote the production of VEGF [26]. The increased VEGF levels can disrupt the structure of occludin and zonula occludens-1 (ZO-1), two membrane proteins that form tight junctions between endothelial cells in the vessel walls, leading to leakage. Moreover, the ability of VEGF to promote mitogen-activated protein (MAP) activity can result in the proliferation of endothelial cells. Both of these phenomena damage and increase the permeability of the retinal vascular walls, leading to macular edema [26]. According to the results of current studies, a characteristic change in the initial retinal changes in DR secondary to the adverse effects of chronic hyperglycemia, AGEs, and local tissue hypoxia is damage to small cells called pericytes. These fibroblast-like cells located in the basal membrane of capillaries are responsible under physiological conditions for supporting vascular endothelial cells to maintain the integrity of the internal blood-retinal barrier (BRB) and regulate capillary blood flow by modeling vascular tone and perfusion pressure [13,37,38]. Notably, microaneurism and neovascularization have been shown to occur more frequently in areas of the retina where pericytes have previously been lost [13,36]. Another factor involved in DME formation is the dysfunction and loss of astrocytes located within the retinal nerve fiber layer (RNFL) and ganglion cell layer (GCL) [39]. Similar to the proteins of the occludin family, these star-shaped cells, with many radiating extensions, play an essential role in establishing tight junctions in the developing retina and maintaining the proper integrity of the vessel walls. A lower density of astrocytes has been found in areas of the retina characterized by an excessively permeable BRB [27].

The association between an increased expression of pro-inflammatory factors and the pathophysiological changes in the retinal microcirculation in the course of diabetes has been demonstrated [26]. Elevated levels of interleukin-1β (IL-1β), interleukin-6 (IL-6), interleukin-8 (IL-8), tumor necrosis factor α (TNF-α), soluble interleukin-2 receptor (sIL-2R), and nitric oxide (NO) correlating with both the presence and the severity of DR were found in the serum of patients with type 2 diabetes [26,40,41]. In another study, increased levels of pro-inflammatory cytokines, including IL-1β, IL-6, IL-8, TNF-α, and neurotrophins, were detected in the vitreous samples of DR patients [42]. Leukostasis, leading to microvessel occlusion and increased adherence of leukocytes to the surface of the retinal vascular endothelium secondary to the overexpression of β2-integrin, intercellular adhesion molecule-1 (ICAM-1), vascular cell adhesion molecule-1 (VCAM-1), and E-selectin, has been found in studies both in people with diabetes and in animals with iatrogenically induced diabetes [26]. Moreover, other studies have shown that activating the Fas pathway (CD95) leukostasis can lead to an increased apoptosis of endothelial cells and damage to the BRB [26]. Additionally, the VEGF-induced overexpression of cytokines, such as monocyte chemoattractant protein-1 (MCP-1), macrophage inflammatory protein-1α (MIP-1α), and macrophage inflammatory protein-1β (MIP-1B), responsible for vascular leakage, stimulating angiogenesis and vascular remodeling, as well as facilitating the infiltration process of the vessel wall by leukocytes and macrophages, has been demonstrated in the vitreous of diabetic eyes [26,43]. Furthermore, under the increased expression of pro-inflammatory cytokines and VEGF, glial cells, including Muller cells and microglial cells, may be activated and, as a result, further produce the pro-inflammatory factors [26].

It is postulated that neurodegenerative processes manifested as ganglion cell loss and retinal thinning may precede the appearance of abnormalities in the retinal microvessels caused by chronic hyperglycemia [Wang]. It is presumed that the key factors responsible for these transformations are the negative influence of oxidative stress and secondary damage to the mitochondria, conditioning the disturbance of the function and structure of retinocytes [26].

## 4. The Role of the VEGF and Ang-Tie Pathways in the Pathogenesis of DME and nAMD

The formation of blood vessels, called angiogenesis, determines the proper development and functioning of the body tissues. However, abnormalities in this process involving the uncontrolled formation of new vessels constitute the pathophysiological background of many diseases, including neoplasms, neoplastic metastases, and pathological conditions with an inflammatory and vascular background [44]. The Ang-Tie pathway, along with the VEGF pathway, are represented by two vascular endothelial-specific signaling pathways of receptor tyrosine kinases (RTKs) that control the development and maintenance of cardiovascular and lymphatic homeostasis [45]. Therefore, their role in the pathogenesis of vascular diseases, including DME and AME, has been widely studied.

### 4.1. VEGF Pathway

The VEGF family includes six VEGF-(A-F) molecules, as well as PlGF-1 and PlGF-2 [33]. VEGF-E is encoded by viruses, while VEGF-F has been found in snake poison; thus, research focuses mainly on four endogenous human molecules, including VEGF-A, VEGF-B, VEGF-C, and VEGF-D. These molecules exert their effects on the body by binding to three types of target cell surface receptors: VEGFR-1 (Flt-1), VEGFR-2 (Flk-1/KDR), and VEGFR-3 (Flt-4) [33]. It is known that individual molecules of the VEGF family have different biological properties and perform different bodily functions. VEGF-A is the predominant mediator of the pro-angiogenic response of all molecules in the VEGF pathway and is present in the human body as sixteen isoforms, of which VEGF-A165 is the best known [46,47,48]. Previous studies have shown that VEGF-A165 is the most specific for ocular tissues and plays a significant role in pathological neovascularization and vascular permeability [34]. The prominent role of VEGF-A is to influence vascular endothelial cells to stimulate their proliferation and migration, while VEGF-B can counteract oxidative stress and inhibit pathological angiogenesis. In contrast, VEGF-C and VEGF-D are mainly regulating the lymphatic system functions, as well as the stimulation of lymphangiogenesis, respectively. VEGF-C and VEGF-D are ligands for the lymphatic growth factor receptor VEGFR-3 [33]. Among the mentioned VEGF receptors, VEGFR-2 is presumed to play a crucial role in the pro-angiogenic, anti-apoptotic, mitogenic, and vascular permeability-promoting responses [11,34]. However, VEGFR-1 showed greater affinity for the VEGF-A165 isoform compared to VEGFR-2; therefore, a fragment of its third VEGF-binding domain was used during the design of the aflibercept molecule in order to improve its effectiveness [11,49].

VEGF-A overexpression is induced by hypoxia, oxidative stress, hypoglycemia, and some pro-inflammatory cytokines, including IL-1β, IL-6, and tumor growth factor β (TGF-β) [49,50]. The primary pathway leading to the upregulation of VEGF-A expression is the HIF-α pathway [Stewart]. Several retinal cells, in the manner of pericytes, RPE cells, astrocytes, neurons, and microvascular endothelial cells, can produce VEGF-A [51]. In addition to playing a significant role in inducing choroidal neovascularization (CNV), VEGF-A is also responsible for increased vascular permeability by promoting the formation of pores in the vascular endothelial cells and the promotion of damage to tight junctions between vascular endothelial cells, leading to an accumulation of intraretinal and subretinal fluid [2,11,52]. Additionally, the increased expression of VEGF signals macrophages for migration into the vascular wall, where they interact with vascular endothelial cells, pericytes, and vascular smooth muscle cells (VSMCs) to induce vascular remodeling. Moreover, macrophages generate VEGF, which causes the further promotion of vascular abnormalities [44,53]. However, the physiological VEGF-A expression in the retina is essential for its proper functioning [54]. VEGF-A is an important factor in the survival of retinal ganglion cells (RGCs), Muller cells, and photoreceptors [47]. Notably, despite the presence of both VEGF-A and VEGFR-2 in CNV lesions in both humans [55] and animals [56,57], isolated VEGF-A overexpression in the absence of favorable pathological factors did not promote CNV formation in a transgenic mouse model study [54]. Thus, in addition to the undisputed involvement of VEGF-A in pathological angiogenesis, it is known that the VEGF pathway is part of a complex mechanism leading to the development of CNV [54].

PlGF is a member of the VEGF family and is binding by VEGFR-1 [58]. It is known that PlGF, in the form of two isoforms, PlGF-1 and PlGF-2, can independent of VEGF stimulate the proangiogenic response [7], and the main factor causing its upregulation is hypoxia [58]. Experiments in a mouse model have shown that physiological angiogenesis can occur without PlGF, while its presence is crucial in abnormal vessel formation [58]. The involvement of PlGF in the development of pathological retinal lesions in both DR and nAMD has also been provided. In vitreous samples of PDR patients, higher levels of PlGF were found [58]. In addition, genetically modified mice deficient in PlGF exhibited a reduced sensitivity to diabetes-induced pathological alternations in the retinal microcirculation, including microvascular damage, pericyte loss, induction of neuronal apoptosis, and BRB damage [49,59]. In contrast, the expression of PlGF, VEGF-A, VEGF-B, VEGFR-1, and VEGFR-2 has been found in removed human neovascular membranes [54]. Moreover, both PlGF-deficient transgenic and PlGF receptor-neutralized mice by an intraperitoneal injection of monoclonal rat anti-mouse Flt-1, a PlGF receptor (VEGFR-1) inhibitor, were resistant to the formation of laser-induced CNV lesions [54].

### 4.2. Ang/Tie Pathway

Ang-Tie receptor pathway is responsible for remodeling both blood and lymphatic vessels during embryonic and postnatal development and maintaining vascular homeostasis by stabilizing mature vessels and regulating their permeability [45]. Additionally, according to the current research results, the Ang-Tie pathway plays an essential role in the modulation of the course of inflammatory processes and participates along with the VEGF pathway in the process of new vessel formation [2]. Therefore, in recent years, attention has been paid to the role of the Ang-Tie pathway in the pathogenesis of retinal vascular diseases, and this issue has been investigated in numerous studies.

Angiopoietins, small endogenous molecules, constitute the family of growth factors involved in the regulation of angiogenesis [60]. To date, four types of angiopoietins, Ang-1, Ang-2, Ang-3, and Ang-4, have been distinguished [61]. Tie receptors were isolated in the early 1990s [60]. These receptors belong to the RTK subfamily and are divided into two subtypes, including the Tie-2 receptor, the primary receptor of the Ang/Tie axis, and the Tie-1 receptor, first discovered in human leukemic cells [60,62]. Tie-1 and Tie-2 receptors are transmembrane receptors, and their extracellular part contains the domains of three molecules, including epidermal growth factor (EGF), fibronectin type III, and immunoglobulin (Ig) [60]. Tie-1 and Tie-2 receptors are located mainly on vascular endothelial cells and some progenitor cells [61]. Angiopoietins are bound by the Tie-2 receptor, while the Tie-1 receptor is classified as an orphan receptor because it has no ligands [60,61]. Previous animal studies have shown that the Tie-1 receptor is vital in the proper development of the circulatory and lymphatic systems and plays a strategic role in the development of pathological vascular system changes, including the progression of atherosclerotic lesions and the growth of tumor vessels [45]. Ang-1 is constitutively expressed in smooth muscle cells, fibroblasts, and pericytes and can activate the Tie-2 receptor through its phosphorylation and thus ensure the maintenance of vascular homeostasis [33]. Ang-1 and Ang-2 are bound by the Tie-2 receptor with similar affinity, competing with each other and exerting a different effect on the Ang-Tie-2 pathway [33]. In vitro studies on bovine vascular endothelium have shown that factors such as hypoxia, hyperglycemia, oxidative stress, and an increase in VEGF-A and fibroblast growth factor (FGF) concentrations may increase Ang-2 expression. In comparison, an increase in Ang-1 concentration reduces Ang-2 synthesis [23,63,64]. Suppression of the Ang-2/Tie-2 pathway may promote the activity of the Ang-1/Tie-2 pathway and the resulting beneficial effects on vascular homeostasis [8]. The Ang-1-dependent stimulation of the Tie-2 receptor located on the endothelial cells of the retinal vessels promotes their stabilization through the recruitment of pericytes and VSMCs [65]. The Ang-1/Tie-2 pathway plays an essential role in the regulation of maturation, maintaining the proper structure and integrity of the vascular endothelium by stabilizing VE-cadherin, the central adhesive molecule of the vascular wall [33]. Activating the Tie-2 receptor, Ang-1, can inhibit nuclear factor ĸβ (NF-ĸβ)/TNF-α-mediated inflammatory response [65] and therefore reduces the destructive influence of pro-inflammatory factors on the inner layer of the vascular wall, contributing to the maintenance of the normal function of blood vessels [2,33,52]. Ang-2, produced by endothelial cells and stored in Weibel–Palade bodies, has an opposite effect to that of Ang-1, and under pathological conditions, it antagonizes Tie-2, inhibiting its phosphorylation, and, as a result, disturbs vascular homeostasis, causing impairment of vascular endothelial function. It damages the BRB and enhances the pro-inflammatory response by abolishing the inhibition of the NF-ĸβ pathway [2,15]. Moreover, under the influence of an increased expression of pro-inflammatory factors, Ang-2 acts as a partial agonist of the Tie-2 receptor, increasing the permeability of the vascular wall and promoting the accumulation of intraretinal fluid leading to retinal thickening [8].

Animal studies have proven that blocking Ang-2 activity may also have a beneficial effect by inhibiting the interaction with the Tie-2 receptor and inhibiting the influence of Ang-2 on integrin signaling. In C57BL/6J mice with diabetes induced by intraperitoneal injection of streptozocin, it was shown that increased levels of Ang-2 in retinal tissue induced pericyte apoptosis under hyperglycemic but not normal serum glucose levels. Interestingly, this phenomenon did not depend on the Tie-2 receptor pathway but was due to the overexpression of integrin-a3 and integrin-β1 in retinal pericytes secondary to hyperglycemia [37]. Another study using the same mouse model revealed the ability of Ang-2 to induce astrocyte apoptosis in hyperglycemic conditions via αvβ5-integrin/GSK-3β/β-catenin pathway with avB5-integrin as the receptor for Ang-2 [27]. Moreover, the Ang-2/Tie-2 pathway interacts with the VEGF pathway, forming vascular abnormalities within the retina [33]. Importantly, studies in a mouse model revealed that Ang-2, through its ability to sensitize the endothelium to the VEGF-A, promotes retinal neovascularization [66]. In contrast, in studies on human epithelial cells, the inhibition of Ang-2 or an increase in Ang-1 concentration could partially reverse the pathological changes induced by VEGF-A, while in a monkey model, the synergistic inhibition of Ang-2 and VEGF-A more counteracted the development of neovascular lesions than separate blocking of these factors [3]. In previous studies, an association between increased levels of VEGF-A and Ang-2 in both diabetic and nAMD eyes has been found. In the vitreous samples of DR patients, increased levels of Ang-2, VEGF, metalloproteinase 2 (MMP-2), metalloproteinase 9 (MMP-9), TGF-β1, and erytrhropoietin (EPO) were detected, while in another study, higher vitreous concentrations of both VEGF and Ang-2 were observed in patients with PDR compared to NPDR [52]. Significantly, the concentration of Ang-2 positively correlated with the levels of VEGF and was associated with the presence of pathological changes, including active neovascularization and retinal traction [52].

Therefore, both Ang-2 and VEGF play an important role in the pathophysiology of DME and nAMD, responsible for neovascularization, vascular leakage, and inflammation—the main pathological processes underlying retinal vascular diseases [67].

## 5. Aflibercept and Faricimab: Molecular Characteristics

**Figure 1:**
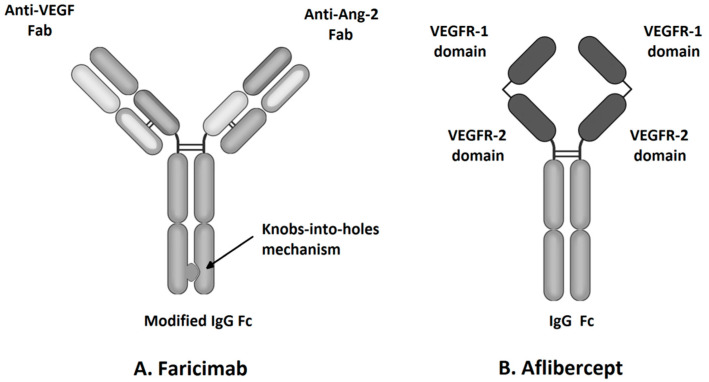
Molecular structure of faricimab and aflibercept.

**Figure 2:**
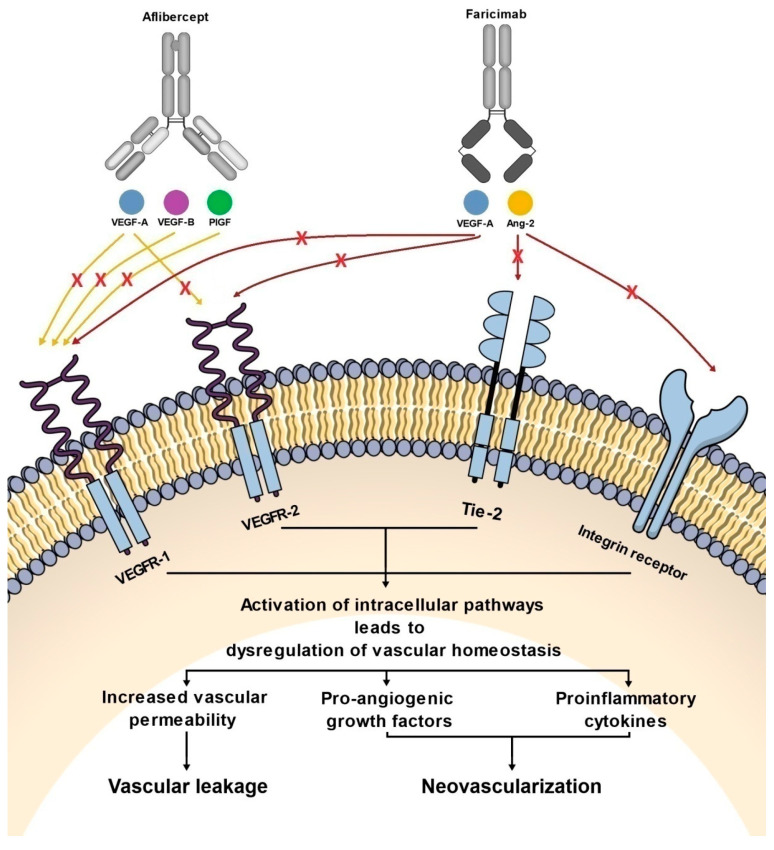
Diagram showing the effect of aflibercept and faricimab on pathways involved in the pathogenesis of nAMD and DME.

Aflibercept, also known as VEGF-Trap Eye, a commonly used intravitreous anti-VEGF agent, was approved by the Food and Drug Administration (FDA) for the treatment of nAMD and DME in 2011 and 2014, respectively (Table 1). VEGF-TRAP was the third anti-VEGF formulation approved by the FDA, after pegaptanib and ranibizumab. Another widely used anti-VEGF drug, bevacizumab, is used intravitreally as an off-label drug [6]. Aflibercept is a recombinant 115 kDa protein composed of the Fc fragment of human IgG1 and the second and third extracellular domains of human VEGFR-1 and VEGFR-2, respectively (Figure 1) [33]. Primarily, this soluble decoy receptor for VEGF was developed for use in oncology as a therapy for solid tumors and tumor metastases. Aflibercept was reformulated to an iso-osmotic form to be administered intravitreally and to reduce systemic exposure associated with the side effects such as hypertension and proteinuria [34]. The structure of the aflibercept molecule allows for competitive binding of all VEGF-A and VEGF-B isoforms with higher affinity than its native receptors, and thus, due to this property, aflibercept is sometimes also called “VEGF Trap-Eye” [8,33]. Aflibercept also can bind PlGF-1 and PlGF-2, other members of the VEGF family; these growth factors have the potential to induce retinal neovascular changes [8,33]. The ability to bind all VEGF family cytokines differentiates aflibercept from other intravitreal anti-VEGF agents, including ranibizumab, bevacizumab, and brolucizumab that inhibit only VEGF-A (Figure 2) [23]. The specific structure of the aflibercept molecule provides it with almost 100 times greater affinity to VEGF-A than ranibizumab and bevacizumab [35]. The half-life of VEGF-Trap Eye after intravitreal administration is 7.1 days, while the duration of clinical activity has been estimated at 2.5 months, which allowed the interval between consecutive intravitreal injections to be extended to 8 weeks [11].

Faricimab, known at the stage of preclinical experiments as RG7716, is the first bispecific monoclonal antibody designed for intravitreal use and was approved for the treatment of nAMD and DME by the FDA in January 2021 (Table 1) [33]. Bispecific heterodimeric antibodies, due to having different light chains in each of the fragment antigen-binding (Fab) regions, are characterized by the possibility of binding two different targets, which is unattainable for traditional monospecific antibodies [69]. These properties were achieved through a knob and hole mechanism between the heavy chains during the drug design process [8] with the CrossMAb CH1-CL technology, first described in 2011 (Figure 1) [69]. Due to its specific structure, the faricimab molecule has one VEGF-binding domain and one Ang-2-binding domain [8], which enables their simultaneous and independent neutralization [33,70]. Notably, previous studies revealed the ineffectiveness of the application during the same injection of a binary composition consisting of Ang-2-binding antibodies, nevascumab, and aflibercept. They showed that using CrossMAb technology, allowing the simultaneous and independent binding of VEGF-A and Ang-2 by the same molecule, is essential to obtain the desired clinical effect (Figure 2) [71]. What is worth emphasizing is that previous studies revealed the ineffectiveness of applying the two-component mixture of Ang-2-binding antibodies, nevascumab, and aflibercept. Further studies using faricimab showed that the use of CrossMAb technology that allows the design of a bispecific antibody capable of the simultaneous and independent binding of VEGF-A and Ang-2 allows the desired clinical effect [71]. In the faricimab molecule, the crystallizable region, also known as an Fc fragment, which binds to cell receptors and complement proteins, has been modified to reduce systemic exposure time and associated side effects by introducing a Triple-A mutation in the FcRn binding region. On the other hand, the reduction in the immune system stimulation was achieved by introducing the P329G LALA mutation, which prevented the binding of the faricimab molecule to Fcγ receptors and eliminated the possibility of an undesirable immune system response after the administration of the drug secondary to interaction with these receptors [69,72,73].

## 6. Summary of Preclinical Studies of Aflibercept and Faricimab

### 6.1. Preclinical Studies of Aflibercept

Before entering clinical trials, aflibercept has been investigated in several preclinical trials. In these experiments, VEGF-Trap showed an ability to inhibit the proliferation of bovine vascular endothelial cells comparable to ranibizumab [74]. Then, the efficacy of aflibercept in inhibiting CNV development was assessed in rodent [4,75] and non-human primate models [76]. In adult rats with CNV induced by subretinal Matrigel injection, two systemic administrations of aflibercept, two days before and six days after Matrigel administration, prevented the development of CNV. In contrast, in CNV developing over ten days, aflibercept prevented the formation of new neovascular lesions and regression of existing lesions, leukocyte infiltration, and the development of fibrosis by inhibiting collagen synthesis [4]. In another study, subcutaneous and intravitreal aflibercept was shown to suppress CNV development in mice with laser photocoagulation-induced BM injury. In addition, the inhibitory effect of aflibercept following subcutaneous administration on CNV formation in transgenic mice with increased VEGF expression induced within the photoreceptor layer was also confirmed. This experiment also demonstrated the protective properties of aflibercept presented against BRB by limiting its damage under increased VEGF concentration in the vitreous, both after the administration of recombinant VEGF and in transgenic mice with the deliberately induced overexpression of VEGF [75]. The ability of VEGF-Trap to prevent and treat laser-induced CNV in cynomolgus monkeys has also been investigated [76]. The results showed that aflibercept was administered weekly intravenously at a dose of 3 mg/kg and 10 mg/kg, as well as intravitreal at a dose of 50 µg, 250 µg, and 500 µg at two-week intervals against laser-induced macular damage, significantly protecting the examined eyes from developing CNV. Moreover, during the study, it was also shown that a single intravitreal injection of aflibercept at a dose of 500 µg reversed neovascular changes induced two weeks earlier by laser and significantly inhibited the formation of advanced vascular leakage sites [76].

### 6.2. Preclinical Studies of Faricimab

In vitro studies have shown that faricimab has the potential to inhibit the destructive effects of VEGF-A and Ang-2 on human vascular endothelial cells. Moreover, in JR5558 mice with spontaneous CNV, the simultaneous inhibition of VEGF and Ang-2 activity significantly reduced vascular permeability and retinal edema, neuronal apoptosis, and macrophage infiltration of neovascular lesions compared to the separate use of VEGF and Ang-2 inhibitors. In the same study, intravitreal faricimab had a more significant potential to reduce leakage lesions than ranibizumab and demonstrated the ability to significantly reduce the concentration of pro-inflammatory IL-6 in the aqueous humor in monkeys with laser-induced CNV [3]. These results were further confirmed in a replication study by Foxton et al. [77].

## 7. Clinical Trials of Aflibercept and Faricimab

### 7.1. Clinical Trials of Aflibercept

#### 7.1.1. Phase I Trials for nAMD: CLEAR-AMD I and CLEAR-IT I

The first clinical trial designed to assess the safety of VEGF-Trap was the randomized, double-masked CLEAR-AMD I [78] trial. In this experiment, 25 nAMD patients received intravenous aflibercept at doses of 0.3 mg/kg, 1.0 mg/kg, and 3.0 mg/kg, and a placebo. The 6-week follow-up showed that the intravenous administration of aflibercept did not significantly affect the best-corrected visual acuity (BCVA). In the groups of patients receiving 1.0 mg/kg and 3.0 mg/kg VEGF-Trap, an improvement in anatomical conditions was observed—a decrease in the central retinal thickness (CRT) and a reduction in SRF. However, the study was terminated due to systemic complications in two patients receiving the 3.0 mg kg injection, one with grade 4 hypertension and another with grade 2 proteinuria [78]. After changing the VEGF-Trap formulation and converting it to an iso-osmotic solution intended for intravitreal injections, the CLEAR-IT I study was performed [Nguyen 79]. In this study, nAMD patients received a single intravitreal injection of aflibercept at 0.05 mg, 0.15 mg, 0.5 mg, 1 mg, 2 mg, and 4 mg, followed by a 6-week follow-up period. During the study, adverse ocular and systemic effects were not observed, and a decrease in CRT (mean change −104.5 µm) was observed in all aflibercept-treated groups with no deterioration in vision in 95% of participants. The greatest improvement in BCVA was seen in the groups receiving 2.0 mg and 4.0 mg aflibercept injections, and it was +13.5 liters (L) compared to a mean improvement of +4.43 L in all patients who received aflibercept (Table 2) [79].

#### 7.1.2. Phase I Trial for DME

The safety and tolerability of aflibercept were assessed in the first phase of clinical trials in five DME patients. Patients with a central subfield thickness (CST) greater than 250 µm measured by OCT and BCVA ranging from 20/40 to 20/320, with an average BCVA of 36 Early Treatment Diabetic Retinopathy Study (ETDRS) L read at 4 m, were included [31]. All participants administered a single injection of aflibercept at a dose of 4.0 mg, and the observation period was six weeks. The CST in the fourth week of the investigation was reduced by an average of 49 µm, while at the end of the observation period, a decrease in CST in four out of five patients was obtained, averaging 34 µm. BCVA improved by an average of +9 L in the fourth week, while four of the five patients achieved a mean visual gain of +3 L at six weeks. During the experiment, no severe ocular and systemic side effects related to the intravitreal administration of aflibercept were found, allowing further studies (Table 3) [31].

#### 7.1.3. Phase II Trial for nAMD: CLEAR-IT-2

In phase II of the clinical trials, aflibercept was evaluated in a randomized, double-masked, CLEAR-IT 2 trial consisting of two phases. The first 12-week phase with five aflibercept dosing regimens, including four 0.5 mg or 2 mg injections every four weeks or two injections—at the baseline and the 12th week of 0.5 mg, 2.0 mg, or 4 mg of aflibercept. The second phase, lasting from 16–52 weeks, consisted of administering the injections “as needed” (pro re nata, PRN) identified at the monthly follow-up visit. The mean central retinal/lesion thickness (CR/LT) reduction was 119 µm and 130 µm, while the BCVA gained +5.7 and +5.3 L at weeks 12 and 52, respectively. The most significant improvement in the vision of nine L was found in patients receiving 2 mg of aflibercept every four weeks in the study’s first phase. Finally, maintenance or improvement in visual acuity was achieved in 73% of patients, of which 22% of participants gained more than 15 L and 8% of participants lost more than 15 L. The results revealed a better visual acuity improvement in patients receiving the four weekly aflibercept injections in the study’s first phase corresponded to the four monthly dosing regimens. Interestingly, in the group receiving 2.0 mg aflibercept in the first phase of the study, the most remarkable improvement in visual acuity was observed, and no patient in this group experienced a decrease in visual acuity above 15 L [81]. In the second phase, with the frequency of injections based on the assessment of disease activity, the patients required an average of two injections, and the lack of disease activity lasting from 16–52 weeks was achieved in 19% of participants (Table 2) [24].

#### 7.1.4. Phase II Trial for DME: DA VINCI Study

The favorable results of the first phase studies on the use of aflibercept in treating DME allowed the initiation of phase II studies. In the DA VINCI study, the efficacy of intravitreal aflibercept injections was compared with that of laser photocoagulation in 221 diabetic patients with center-involving DME. VEGF-Trap was administered in four regimens consisting of 0.5 mg and 2 mg aflibercept injections every 4 weeks, as well as three 4 weekly injections of 2 mg aflibercept with continuation every 8 weeks or as needed (PRN). The outcomes of the 24-week observation revealed a significant benefit of aflibercept in improving BCVA and CRT corresponded to laser treatment. Participants treated with aflibercept gained an average of +8.5 to +11.4 L compared to +2.5 L in the laser group. A total of 93% of patients in the aflibercept arm maintained or improved their BCVA compared to the laser group; an increase in BCVA over 15 L was marked in 34% and 21% of subjects treated with VEGF-Trap and laser photocoagulation, respectively. Additionally, aflibercept also showed more significant potential in reducing CRT, ranging from −127.3 µm to −194.5 µm in the aflibercept arms, while a reduction of −67.9 µm was observed in the laser group [29]. The beneficial effects were maintained at week 52. An increase in the BCVA greater than 15 L was observed in 23.8–45.5% of patients receiving aflibercept and in only 11.4% of the laser group, with an average improvement of +9.7 to +13.1 L in patients treated with aflibercept compared to a loss of an average of −1.3 L in the laser group. The tendency for the decrease in the mean CRT value in aflibercept regimens continued. CRT reduction in the aflibercept groups ranged from −165.4 µm to −227.4 µm. On the other hand, laser photocoagulation did not maintain the decrease in the mean CRT value obtained at week 24. After one year, a mean reduction in CRT of 58.4 µm was observed in the participants treated with laser. Therefore, the results of the 52-week follow-up showed the stability of the improvement in vision and anatomical parameters in patients treated with aflibercept in various regimens. Similar to the phase I studies, aflibercept was well tolerated, and no significant complications were directly related to the drug effect. Adverse effects were typical of patients undergoing the intravitreal injection procedure and occurred with a similar frequency among the studied groups (Table 3) [84].

#### 7.1.5. Phase III Trials for nAMD: VIEW 1 and VIEW 2

In phase III clinical trials, in the twinning studies VIEW 1 and VIEW 2, including a total of 2419 nAMD patients with active, subfoveal CNV, the efficacy and safety of aflibercept dosed at 0.5 mg or 2 mg every four weeks and 2 mg every eight weeks, after the initial three monthly doses, was compared with 0.5 mg ranibizumab dosed in four-week intervals. The 52-week results of the VIEW-1 and VIEW-2 studies demonstrated a non-inferior effect in the maintenance and improvement of visual acuity and terms of anatomical parameters, including reduction in CRT, as well as the presence of SRF and IRF in all groups in the aflibercept arm compared to ranibizumab [82]. The study continued for another year; however, the dosing regimen in the study groups was modified to a mandatory quarterly injection with possible additional injections of PRN in the event of an increase in disease activity during the monthly follow-up visits. The outcomes after 96 weeks showed a slight decrease in both BCVA and anatomical parameters in patients receiving ranibizumab and aflibercept compared to the 52-week results; however, comparative group analysis showed similar functional and anatomical effects between the study groups with a concurrently available lower injection frequency in patients treated with aflibercept (Table 2) [29].

#### 7.1.6. Phase III Trials for DME: VIVID and VISTA

VIVID and VISTA were two parallel phase III studies assessing the effectiveness of intravitreal aflibercept injections compared to laser treatment in DME [35]. Eight hundred seventy-two diabetic patients with DME affecting the central part of the macula were enrolled. Aflibercept was provided in two schedules—a dose of 2 mg every four weeks or 2 mg every eight weeks after the initial five monthly injections. The third group included patients treated with macular laser photocoagulation at baseline and then as needed with a minimum interval of 16 weeks between laser sessions. At week 52 of the VISTA study, the mean BCVA improvement in the aflibercept arms was +12.5 and +10.7 L, respectively, while patients in the laser group receiving sham injections gained +0.2 L. Similar results were seen in the VIVID study, where the mean BCVA of patients receiving aflibercept injections increased by +10.5 and +10.7 L compared to +1.2 J in laser-treated patients. A reduction in CRT −185.9 µm and −183.1 µm, as well as −195.0 µm and −192.4 µm in the aflibercept groups in the VIVID and VISTA studies, was observed while the laser provided an average reduction in CRT of −73.3 µm and −66.2 µm, respectively [35].

The study was extended for three years, and the results were published by Heier et al. in 2016 [24]. At 148 weeks, the beneficial effect achieved at 52 and 100 weeks on aflibercept regimens was maintained. In VISTA, the improvement in BCVA was +10.4 and +10.5 L, compared to +1.4 L in patients treated with laser photocoagulation. The percentage of subjects with an increase in BCVA over 15 L was 42.9%, 35.8% in patients receiving injections, and 13.6% in the laser group. Similarly, in the VIVID study, at 148 weeks, there was a sustained improvement in vision of +10.3 and +11.7 L compared to baseline in the aflibercept arms compared to +1.6 L in the laser arm. The percentage of patients with sustained improvement in the BCVA above 15 L was 41.2%, 42.2%, and 18.9%, respectively. Additionally, aflibercept allowed for significantly more frequent improvement above two steps in the DRSS scale compared to laser therapy, 29.9% and 34% vs. 20.1% in VISTA, as well as 44.3% and 47.8% vs. 17.4% in the VIVID study (Table 3) [24].

### 7.2. Clinical Trials of Faricimab

#### 7.2.1. Phase I Clinical Trial

In phase 1 clinical trials, a single injection of 0.5 mg, 1.5 mg, 3 mg, and 6 mg faricimab was administered to 24 nAMD patients with visual acuity ranging from 20/40 to 20/400. Patients who received at least three anti-VEGF injections in the prior six months and had shown CNV on fluorescein angiography (AF) or fluid on OCT despite treatment were included. Furthermore, three patients in the 3 and 6 mg groups received two consecutive injections of the same dose. Among patients receiving single and multiple injections at a dose of 6.0 mg, significant improvements in BCVA by +7 and +7.5 L and a reduction in CRT by −42 µm and −117 µm were observed. After three doses of 3.0 mg faricimab, no noteworthy change in BCVA and CRT was observed compared to baseline measurements (Table 4) [85].

#### 7.2.2. Phase II Clinical Trials for nAMD: AVENUE and STAIRWAY

The STAIRWAY study was conducted in a population of 79 nAMD patients over 56 weeks. Participants received 0.5 mg ranibizumab every 4 weeks or 6 mg faricimab every 12 or 16 weeks after four previous monthly injections of faricimab at the same dose. Patients receiving faricimab every 16 weeks were assessed for signs of disease activity at 24 weeks, and if they were present, they were given further injections every 12 weeks, and in the absence of alarming symptoms, continued treatment as before. The primary endpoint was BCVA change at week 40 of the study. The results showed that faricimab administered at 12- and 16-week intervals maintained visual acuity and improved anatomical parameters observed in macular OCT not inferiorly to ranibizumab administered every month (Table 4) [73].

In the AVENUE study, 263 treatment-naive nAMD participants were treated with five different regimens, including injections of ranibizumab at 0.5 mg every four weeks, faricimab at 1.5 mg every four weeks, faricimab at 6.0 mg every four weeks, every four weeks, as well as 6 mg of faricimab for the first 12 weeks with subsequent injections every 8 weeks and a mixed regimen of two injections of ranibizumab at a 0.5 mg dose followed by 6.0 mg injections of faricimab every 4 weeks. The results of the 36-week experiment revealed substantial difference in mean BCVA between the analyzed regimens. Likewise, the study groups showed equivalent improvement in anatomical parameters—reduction in CST and CNV and leakage areas. This study demonstrated that faricimab dosed at longer intervals is an effective alternative to the 4 weekly ranibizumab injections at a 0.5 mg dose (Table 4) [65].

#### 7.2.3. Phase II Clinical Trial for DME: BOULEVARD

In the BOULEVARD study, 168 DME patients were treatment-naive, and 61 DME patients had a history of anti-VEGF treatment. Patients previously untreated with anti-VEGF agents received 1.5 mg or 6 mg faricimab and 0.3 mg ranibizumab, while non-treatment naive patients received 6 mg faricimab and 0.3 mg ranibizumab. All groups were injected monthly for the first 20 weeks, and then patients were followed up for 16 weeks. In the treatment-naive group of patients, a visual improvement of +13.9 in the 6.0 mg faricimab group and +11.7 L in the 1.5 mg faricimab group was observed compared to +10.3 L in the 0.5 mg ranibizumab arm. Similarly, in patients with a history of anti-VEGF treatment, a BCVA gain of +9.6 L in the 6.0 mg faricimab arm and +8.3 L in subjects treated with aflibercept was observed. In both treatment-naive and non-treatment naive patients, a more marked improvement in anatomical parameters and DRSS scores in faricimab regimens was noted compared to the aflibercept arm (Table 5) [15].

#### 7.2.4. Phase III Clinical Trials for nAMD: TENAYA and LUCERNE

Faricimab has been compared with aflibercept in the treatment of nAMD and DME in phase 3 clinical trials. Two multicenter, randomized, phase III studies, TENAYA and LUCERNE, were conducted to evaluate and compare the use of intravitreal injections of 6 mg faricimab every 8, 12, or 16 weeks depending on individual disease activity assessment and 2 mg aflibercept in nAMD patients. The above studies were conducted on 1.329 nAMD treatment-naive patients over 50 years. The primary endpoint of both studies was the BCVA change at 48 weeks. The results showed that, after one year, faricimab dosed every four months allowed for non-inferior visual improvement compared with aflibercept at week 48% in 45.7% and 44.9% of patients in TENAYA and LUCERNE, respectively. Moreover, in these studies, 34.0% and 32.9% of patients treated with every 3-month injections of faricimab achieved similar results to the aflibercept group. Thus, during the first year of treatment, 80% of patients in the TENAYA study and 78% of patients in the LUCERNE study could receive injections less frequently than every two months as used in the aflibercept regimen. Additionally, the examined groups revealed comparable improvements in anatomical parameters, including CST reduction, CNV lesions, and leakage size (Table 4) [16].

#### 7.2.5. Phase III Clinical Trials for DME: YOSEMITE and RHINE

To compare the effects of faricimab and aflibercept treatment in DME patients, two randomized, multicenter studies called YOSEMITE and RHINE were conducted in a group of 940 and 951 patients, respectively. The dosing regimen consisted of 6.0 mg injections of faricimab at 8-week intervals, 6.0 mg injections of faricimab at personalized disease activity intervals, and a group of patients receiving 2.0 mg aflibercept at 8-week intervals. The primary endpoint was BCVA change after one year of treatment. Notably, 52.8% and 51% of patients receiving faricimab every 16 weeks in YOSEMITE and RHINE achieved comparable results in BCVA compared to aflibercept dosed every eight weeks. In addition, 21% and 20.1% of patients were able to administer faricimab every three to twelve weeks to achieve non-inferior visual acuity compared to patients in the aflibercept arm. Overall, in 73.8% of patients in the YOSEMITE study and 71.1% in the RHINE study, treatment with faricimab achieved comparable BCVA gain to treatment with aflibercept while reducing the frequency of injections. The analysis of anatomical parameters also showed similar effects of both agents. A comparison of CST and the amount of IRF showed a more significant reduction in patients treated with faricimab in both studies. Further, in both groups of patients receiving faricimab, a higher percentage of the absence of clinically significant macular edema was observed in OCT (CST less than 325 µm). Similar to the previous studies, faricimab was well tolerated, and the incidence of AEs was similar between the examined groups (Table 5) [17].

## 8. Summary of Pre-Clinical and Clinical Trials of Aflibercept and Faricimab

The promising results of pre-clinical studies with aflibercept and faricimab limiting CNV and vascular leakage in animal models led to the initiation of clinical trials. In the phase I CLEAR-AMD study, aflibercept was administered intravenously in nAMD patients. This experiment was terminated due to side effects observed in participants, including grade 4 hypertension and grade 2 proteinuria [78]. Then, aflibercept was reformulated to an iso-osmotic solution intended for intravitreal injections. The safety and tolerability of intravitreal aflibercept were evaluated in the CLEAR-IT study. There were no serious adverse ocular and systemic events [79]. The further phases of clinical trials of both aflibercept and faricimab showed a similar safety profile for these two agents.

In phase 3 clinical trials for faricimab, aflibercept in the second year was administered at 8-week intervals, while integrated post hoc analysis of VIEW 1 and VIEW 2 trials showed that, among patients receiving aflibercept every four weeks and eight weeks in the first year, 53.9% and 47.9% of participants in the second year maintained satisfactory management of disease activity with injections at 12-week or longer intervals [86]. Additionally, a recent study showed that 45% of patients did not respond to other anti-VEGF agents, including 0.5 mg ranibizumab, 1.25 mg bevacizumab, or 2.0 mg aflibercept, may obtain benefits with 4 mg of aflibercept administered in 4-week intervals. It is worth emphasizing that in the final point of this experiment, 39% of patients had a complete abolishment of the fluid presence. Furthermore, 4 mg of aflibercept dosing at four-week intervals significantly improved anatomical parameters, including central foveal thickness (CFT), maximum foveal thickness (MFT), and sub-RPE fluid. The 4 mg dose of aflibercept was well tolerated and was not without severe AEs [87]. Phase 3 trials, PHOTON and PULSAR, are currently underway. In those studies, aflibercept 8 mg is compared to aflibercept 2 mg at 12- and 16-weeks intervals in patients with DME and nAMD [88,89].

The effectiveness of a high dose of aflibercept compared to faricimab remains to be investigated. This analysis may be especially interesting in the group of patients with refractory nAMD and DME, as well as in a group of patients with markers that may predict a worse response to anti-VEGF treatment. It was postulated that hidden CNV and the presence of PED are responsible for poor response to anti-VEGF drugs in nAMD patients [90]. While symptoms of macular ischemia in AF, including large avascular zone and the high number of microaneurisms are more commonly observed in DME patients with an unsatisfactory effect of anti-VEGF therapy [91]. Additionally, studies comparing the efficacy of faricimab with other anti-VEGF agents in a group of patients with expected high activity of both the VEGF and Ang-2/Tie-2 pathways, e.g., in a population with comorbid diabetes and nAMD, may be of particular interest.

## 9. Advances in the Treatment of nAMD and DME

In recent years, therapy with anti-angiogenic agents has been the most dynamically developed course of treatment for nAMD and DME. However, other therapeutic approaches for these conditions are also being deeply investigated [92]. The era of anti-angiogenic agents allowing for injections of less than eight weeks began with the introduction of brolucizumab. This single-chain antibody fragment (scFv) binds all VEGF-A isoforms with greater affinity than ranibizumab and aflibercept [93,94]. Additionally, the low molecular weight of the molecule amounting to 26 kDa determines good retinal penetration [94]. The FDA approved brolucizumab for the treatment of nAMD and DME in 2019 and 2021, respectively [22,93]. Another low molecular weight molecule is Abicipar Pegol. This agent mimics molecules containing antibody fragments belonging to designed ankyrin repeat proteins (DARPins), non-monoclonal antibodies characterized by high VEGF-A-binding affinity and low molecular weight (36 kDa), which determines good penetration into the retina [95]. These properties, similar to brolucizumab, allowed for the extension of the intervals between injections to 12 weeks in the PALM study (phase II study) in DME patients and the SEQUOIA, CEDAR, and MAPLE studies (phase III studies) in nAMD patients [93,94]. However, despite the promising anatomical and functional results from the 12-week injection regimen [96], a high percentage of sterile endophthalmitis was noted during phase III clinical trials in nAMD patients [95], hence the 2020 FDA rejected abicipar pegol as an nAMD drug.

KSI-301 is an anti-VEGF agent designed on the antibody biopolymer conjugate (ABC) platform with a total molecular weight of 950 kDa. These properties determine the extension of the ocular half-life, which, according to the results of the phase II studies GLEAM and GLIMMER, allows for extending the intervals between successive injections to 16–20 weeks [92,97]. Hence, this drug is a promising therapeutic option for patients with nAMD and DME. In addition to inhibiting the activity of the VEGF-A pathway, the inhibition of other VEGF family pathways is also under investigation. The newly created molecule, named at an early stage OPT-302, can neutralize VEGF-C and VEGF-D molecules. This new therapeutic method, providing a supplement to VEGF-A therapy, showed promising results in Phase Ib/2a clinical trials in DME patients when comparing hybrid therapy with aflibercept versus aflibercept monotherapy and in phase II clinical trials in nAMD patients where the efficacy of combination therapy was compared OPT 302 and ranibizumab monotherapy ranibizumab. In nAMD patients, phase III ShORe and COAST studies comparing the effectiveness of OPT-302 in combination with ranibizumab and aflibercept versus monotherapy with those monoclonal antibodies have been launched [92].

An alternative that avoids intravitreal injections in patients with retinal vascular diseases is port delivery systems (PDSs) that ensure a slow release of the drug molecule. Currently, PDS-releasing ranibizumab require filling with a transconjunctival injection every six months (phase III clinical trials PAGODA and PAVILION in DME) and containing biodegradable microparticles of sunitinib-malate, GB-102 (phase IIb clinical trial ALITISSIMO in nAMD), which can provide an extend the intervals between successive drug administration by up to 6 months. Additionally, studies on GB-103 administered every 12 months have been initiated. However, before the widespread use of PDSs in clinical practice, doubts, especially related to the effect of surviving VEGF pathway inhibition on the retinal tissue, and the consideration of the benefit-risk balance of the need for surgical port implantation procedure, which carries the risk of complications related to the surgery, especially intravitreal hemorrhage, should be considered [92].

Another direction of nAMD therapeutic methods development is stem-cell-derived RPE cell transplantation to regenerate the RPE layer responsible for photoreceptors’ proper functioning and survival. This method has been used in humans, with success expressed as a significant improvement in vision. However, its widespread use limits the efficiency of obtaining an appropriate amount of RPE cells from human pluripotent stem cells (hPSC), as well as the appropriate modulation of the immune response and prevention of inflammatory processes after delivery of the cells to the eye. These problems must be addressed to further develop this therapeutic method [98].

Gene therapy, originally developed as a treatment method for inherited retinal diseases, is nowadays of interest as a potential nAMD treatment method for providing chronic VEGF blockade. RGX-314, ADVM-022, and ADVM-032 are currently the leading and most promising gene therapies under investigation. RGX-314 is based on the adeno-associated virus serotype 8 (AAV8) vector and, when captured by retinal cells, produces a fragment of the ranibizumab-like monoclonal antibody that binds and neutralizes VEGF-A. RGX-314 can be delivered into the suprachoroidal space or as a subretinal injection during vitrectomy [92]. ADVM-022 and ADVM-032 are also based on the AAV vector and contain aflibercept and ranibizumab coding sequences [99], respectively. Intravitreal ADVM-022 has been selected for further studies, and the OPTIC phase I clinical trial results are now pending. Other currently investigated directions of gene therapy in the treatment of nAMD include enhancement of angiostatin and endostatin expression, as well as modulation of the complement cascade (AAV-CD59) activity [100].

Intravitreal steroids are also an effective and safe therapeutic option in DME, and their efficacy is exceptionally high in patients with chronic macular edema. Their benefit is that steroids can be safely used during pregnancy, contrary to anti-VEGF drugs. In addition to the intravitreal injections of triamcinolone, the currently used biodegradable intravitreal implants of dexamethasone and fluocinolone ensure a slow release of those steroids over 4 and 36 months, respectively [101].

## 10. Conclusions

To summarize, both aflibercept and faricimab are valuable therapeutic options for patients with retinal vascular diseases due to their extended range of action that includes molecules other than VEGF-A. Intravitreal anti-angiogenic drugs, characterized by a prolonged clinical activity after intravitreal administration, allowing for proper control of the disease activity with a simultaneous lower injection frequency, reduce the treatment burden in patients and treatment centers. Due to the alarming prognosis of the increasing incidence of both nAMD and DME, developing new therapeutic approaches for nAMD and DME using advanced pharmacotherapy targeting alternative pathways is critical in the fight against the global epidemic of blindness and visual impairment caused by DME and nAMD. The novel ocular antibody, faricimab, due to its ability to directly affect the two different signaling pathways that play a significant role in retinal vascular dysfunction, the VEGF and Ang/Tie-2 pathways, marks the beginning of a new era in the use of bispecific antibodies in intravitreal therapy. Due to its dual mode of action, faricimab may have a particular therapeutic benefit in patients not responding to the available intravitreal therapy; however, these considerations must be confirmed in further studies.

## Figures and Tables

**Table 1:** Comparison of general information and pharmacological properties for aflibercept and faricimab.

Drug’s Name		Aflibercept	Faricimab	Reference
Other used name (s)		VEGF-Trap Eye	RG7716	[3,11]
Molecular weight		115-kDa	150-kDa	[3,11]
Molecular structure		Binding domains of human VEGFR-1 and VEGFR-2 combined with the constant Fc domain of human IgG1	Two different light chains and two different heavy chains combined with modified human IgG1	[3,16]
Binding molecules		VEGF-A, VEGF-B, PlGF-1, PlGF-2	VEGF-A, Ang-2	[11]
Intravitreal dose used in clinical practice		2 mg	6 mg	[3,11]
Date of FDA approval for:	nAMD	2011	2022	[11,68]
	DME	2014	2022	[11,68]
Registered indications		nAMD, DME, myopic choroidal neowascularization, macular edema secondary to retinal vein occlusion	nAMD, DME	[11,16]

Abbreviations: Ang-2, angiopoietin-2; DME, diabetic macular edema; FDA, Food and Drug Administration; nAMD, neovascular age-related macular degeneration; PlGF-1, placental growth factor 1; PlGF-2, placental growth factor 2; VEGF-A, vascular endothelial growth factor A; VEGF-B, vascular endothelial growth factor B; VEGFR-1, vascular endothelial growth factor receptor-1; VEGFR-2, vascular endothelial growth factor receptor-2.

**Table 2:** Summary of clinical trials assessing the use of aflibercept in the treatment of nAMD.

Trial Name	Phase	Number of Participants	Study Design	Primary and Secondary End Measurements	Explanatory Comment ^a,b^	Reference
CLEAR-AMD 1	I	25 nAMD patients	Single intravenous aflibercept administration at a dose: (1) 0.3 mg/kg; (2) 1.0 mg/kg, (3) 3.0 mg/kg with 4-week observational period and then 3 doses in 2-week intervals (4) Placebo	Assessment of the safety, pharmacokinetics, and biological activity of intravenous aflibercept	- 2 of 5 participants that received a 3.0 mg/kg dose presented toxicity symptoms - One patient developed grade 4 hypertension, and another one had grade 2 proteinuria - BCVA remains unchanged - CRT decreased −10%, −66%, −60%, and −12% in examined groups, respectively - 1.0 mg/kg of aflibercept was a maximally tolerated dose - Patients from the 3.0 mg/kg regimen were withdrawn from the experiment, and the study was terminated	[78]
CLEAR-IT 1	I	25 nAMD patients	Single IVA injection in doses: (1) 0.05 mg; (2) 0.15 mg; (3) 0.5 mg; (4) 1.0 mg; (5) 2 mg; (6) 4 mg. vs. placebo with 6-week follow-up	Safety assessment	6-week results: - The mean change of BCVA was +4.43 L (in the 2 mg and 4 mg groups, BCVA gained +13.5 L) - The mean decrease in CRT was −104.5 µm During the study, all dosing regimens had no ocular AEs or SAEs.	[79]
CLEAR-IT 2	II	159 nAMD patients with subfoveal CNV	Phase 1 (fixed-dosing): 1–12 weeks Five dosing regimens of IVA: (1) 0.5 mg every 4 weeks (2) 2 mg every 4 weeks (3) 0.5 mg every 12 weeks (4) 2 mg every 12 weeks (5) 4 mg every 12 weeks Phase 2: 12–52 weeks Further PRN dosing in all regimens examined in phase 1	Phase 1 Primary: - Change in CL/LT at week 12 Secondary: - Change in BCVA - Proportion of patients that gained and lost 15 or more litres Phase 2 - Change in CR/LT - Change in CNV lesion size - Mean change in BCVA - Proportion of patients that gained and lost 15 or more L - Time for the first PRN injection - Reinjection frequency - Safety	12-week results: - The mean decrease in CR/LT in IVA regimens was −119 µm (with the most improvement in (1) and (2) groups - BCVA gain in IVA groups was +5.7 L (with the >8 L gain in (1) and (2) groups) - 98% of patients avoided −15 or more L decrease in BCVA - 18.5% of individuals gained 15 or more liters There were no significant ocular ad systemic AEs. 52-week results: - Mean decrease in CR/LT in IVA regimens was −130 µm (with the most improvement in (1) and (2) groups - CNV lesion size regression was −2.21 mm - BCVA gained in IVA groups was +5.3 L (the most marked improvement was +9 L in (2) group) - Mean 2 injections were administered in IVA regimens - Mean time to the first injection in phase 2 was 129 days - 19% of participants did not need injections in phase 2, and 45% of participants received 1 or 2 injections - 92% of participants avoided −15 or more liters decrease in BCVA - 22% of patients gained 15 or more liters IVA was well tolerated, and ocular as well as systemic AEs were typical to those previously reported in other anti-VEGF agents	[80,81]
VIEW-1 and VIEW-2	III	2419 nAMD patients with active, sub-foveal CNV	Four dosing regimens in the first 52 weeks: (1) 0.5 mg IVA every 4 weeks (2) 2 mg of IVA every 4 weeks (3) 2 mg of IVA every 8 weeks after 3 injections at 4-week intervals (4) 0.5 mg IVR every 4 weeks 52–96 weeks: PRN dosing regimen in all groups	Primary - Comparison of IVA dosing regimens with the IVR group (testing for non-inferiority—with 10% margin) in the proportion of participants with vision loss <15 L at week 52 and 96 week Secondary: - Change in BCVA - Proportions of patients with +15 letter gain - Change in NEI VFQ-25 score - Change in CNV lesions area - Change in CRT and the persistence of fluid	52-week results of VIEW 1: - There were of participants with vision loss <15 L 94.4% in IVR arm vs. 95.1%, 95.9%, 95.1% in IVA regimens - The mean change in BCVA was +8.1 ± 15.3, +10.9 ± 13.8, +6.9 ± 13.4, and +7.9 ± 15.0 L, respectively - The mean CRT decreased by −116.8 ± 109.0 µm, −116.5 ± 98.4 µm, and −115.6 ± 104.1 µm in IVA groups, while in the IVR regimen, the mean change was −128.5108.5 µm - Change in the NEI VFQ-25 score was 4.9 ± 14.0 in the IVR arm vs. 6.7 ± 13.5, 4.5 ± 11.9, and5.1 ± 14.7 in patients who received IVA - The mean decrease in CNV lesion area was 4.2 ± 5.6 mm^2^ in participants following IVR and 4.6 ± 5.5, 3.5 ± 5.3, and 3.4 ± 6.0 in patients treated by IVA - There were no SRF or IRF in 63.6% of patients treated by IVR compared to 64.8%, 56.7%, and 63.4% of patients receiving IVA 52-week results of VIEW-2: - 94.4% of patients followed IVR loose less than 15 L compared to 95.6%, 96.3%, and 95.6% IVA regimens - The mean change in BCVA was +9.4 ± 13.5 in the IVR group vs. +7.6 ± 12.6, +9.7 ± 14.1, and +8.9 ± 14.4 in IVA regimens - The mean CRT reduced −138.5 ± 122.2 µm in participants that received IVR vs. −156.8 ± 122.8 µm, −129.8 ± 114.8 µm, and −149.2 ± 119.7 µm in IVA groups -The mean change in the NEI VFQ-25 score was 6.3 ± 14.8 in the IVR arm vs. 4.5 ± 15.0, 5.1 ± 13.7, and 4.9 ± 14.7 mm^2^ in patients who received IVA - Decrease in CNV lesion area was 4.2 ± 5.9 mm^2^ in participants following IVR and 6.0 ± 6.1, 4.2 ± 6.1, and 5.2 ± 5.9 mm^2^ in patients treated by IVA SRF and IRF were absent in 60.4% of participants in the IVR arm vs. 80.3%, 63.9%, and 71.9% of patients in the IVA groups 96-week combined results of VIEW-1 and VIEW-2: - 91.6% of participants lose <15 L in IVR arm vs. 92.2%, 91.5%, and 92.4% in IVA regimens - The mean BCVA gained +7.9 L in the IVR arm vs. +7.6, +6.6, and +7.6 L in IVA regimens - The mean change in CRT was −118 µm in the IVR arm vs. 113, 128, and 133 µm in IVA regimens - SRF and IRF were absent in 45.5% of participants in the IVR arm vs. 54.4%, 44.6%, and 50.1% of patients in the IVA groups - 73.5/26.5% vs. 86.0%/14.0% vs. 76.0%/24.0% vs. 84.1%/15.9% patients received less/more than 6 injections in PRN phase in IVR, and IVA groups, respectively A similar frequency of AEs and SAEs was observed between examined groups during the first and PRN phase of the study.	[29,82]

Abbreviations: AEs, adverse events; anti-VEGF, anti-vascular endothelial growth factor; BCVA, best-corrected visual acuity; CNV, choroidal neovascularization; CRT, central retinal thickness; CR/LT, central retinal/lesion thickness; IRF, intraretinal fluid; IVA, intravitreal aflibercept; IVR, intravitreal ranibizumab; L, liters; nAMD, neovascular age-related macular degeneration; NEI VFQ-25, national eye institute visual function questionnaire; PRN, pro re nata (as needed); SAEs, serious adverse events; SRF, subretinal fluid. ^a^ BCVA was measured according to the ETDRS protocol. ^b^ The results for each group are provided in the order described in the “Study Design” Section.

**Table 3:** Summary of clinical trials assessing the use of aflibercept in the treatment of DME.

Trial Name	Phase	Number of Participants	Study Design	Primary and Secondary Outcomes	Explanatory Comment ^a,b^	Reference
-	I	6 patients with diabetic macular edema	Single 4 mg IVA injection with 6 weeks follow-up	Safety and tolerability of IVA Changes in BCVA and change in CRT at four and six weeks	4-week results: - The mean BCVA improvement +9 L - Mean CST reduction −49 µm 6-week results: - The mean BCVA improvement +3 L achieved in 4 of 5 participants - The mean CST reduction −34 µm achieved in 4 of 5 participants There were no ocular and systemic side effects	[31]
DA VINCI	II	221 patients with type 1 or type 2 diabetes presented center-involved DME	5 treatment regimens: (1) Injection of 0.5 mg IVA every 4 weeks (2) Injection of 2 mg of IVA every 4 weeks (3) Injection of 2 mg IVA for the first 3 months, and then in 8-week intervals (4) Injection of 2 mg IVA for the first 3 months, and then PRN treatment (5) Macular laser photocoagulation at baseline and the as needed (with minimum 16-week intervals) + sham injections every 4 weeks	Primary: - Improvement in BCVA at 24 weeks - Assessment of safety and tolerability of IVA injections in DME eyes Secondary: - Change in CRT value from baseline up to 24 weeks - Improvement in BCVA and CRT at 52 weeks - Proportions of eyed gained +15 L at 24 and 52 weeks - Number of focal lasers sessions	24-week results: - BCVA improvement in IVA regimens ranged from +8.5 to +11.4 vs. +2.5 L in the laser + sham injection arm - Improvement of BCVA in IVA vs. laser arms, respectively: 93% vs. 68% of patients gained +0 L 64% vs. 32% of patients gained +5 L 34% vs. 21% of patients gained +15 L - The mean decrease in CRT values in the IVA regimens ranged from −127.3 µm to −194.5 µm vs. 67.9 µm in the laser + sham injections arm - Mean < 1.7 focal laser sessions were performed in the laser group from baseline up to 24 weeks 52-week results: - BCVA improvement in IVA regimens ranged from +9.7 to +13.1 L vs. −1.3 L in the laser and sham injection arm - From 23.8% to 45.4% vs. 11.4% of patients gained +15 L in IVA vs. laser arms, respectively - From 45% to 71% vs. 30% patients gained +10 L in IVA vs. laser arms, respectively - The mean decrease in CRT values in the IVA regimens ranged from −165.4 µm to −227.4 µm vs. 58.4 µm in the laser + sham injections arm - <1 and 2.5 focal laser sessions were performed in IVA groups vs. laser arm from 24 to 52 weeks - Baseline DRSS value ranged from 31% to 64% in IVA arms vs. 12% in the laser regimen, and at 52 weeks, DRSS value ranged from 0% to 14% in IVA groups vs. 24% in the laser arm Ocular adverse effects were mild and typical for intravitreal injections, while systemic adverse effects were not directly associated with the drug action.	[83,84]
VIVID and VISTA	III	872 patients with type 1 or 2 diabetes presented center involved DME	3 treatment regimens: (1) Injection of 2 mg of IVA every 4 weeks (2) Injection of 2 mg IVA for the first 5 months, and then in 8-week intervals (3) Macular laser photocoagulation at baseline and the as needed (with minimum 16-weeke intervals) + sham injections every 4 weeks	Primary: - Improvement in BCVA at 52 weeks Secondary: - Mean change in CST at 52 weeks - Proportions of eyed gained +15 at 52 weeks	52-week results: VIVID: - The mean change of BCVA in IVA arms was +10.5 and +10.7 compared to +1.2 L in the laser group - Participants with a BCVA improvement of +15 L or more accounted for 32.4% and 33.3% in IVA groups vs. 9.1% in the laser arm - The mean change in CST was −195 µm and 192.4 µm in IVA regimens vs. 66.2 µm in the laser group VISTA: -The mean change of BCVA in IVA arms was +12.5 and +10.5 compared to +0.2 L in the laser arm - There were 41.6% and 31.1% vs. 7.8% of participants who gained that +15 L - The mean change in CST was −185.9 µm and 183.1 µm in IVA regimens compared to −73.3 µm in the laser arm 100-week results: VIVID: - The mean change of BCVA in participants following IVA was +11.4 and +9.4 vs. +0.7 L in individuals treated by the laser photocoagulation - 38.2% and 31.1% of patients gained 15 or more L in IVA regimens vs. 12.1% in the laser arm - The mean change in CST was −211.8 ± 150.9 µm and −195.1 ± 141.7 µm in IVA regimens vs. −85.7 ± 179.3 µm in the laser group - 29.3% and 32.6% of participants achieved at least 2-step improvement in DRSS score compared to 8.2% in the laser arm VISTA: - The mean change in BCVA in IVA regimens was +11.5 and 11.1 vs. +0.9 L in the laser arm - 38.3% and 33.1% of participants in IVA groups gained 15 or more L vs. 13.0% in the laser group - CST changed −191.4 ± 180.0 µm; −191.1 ± 160.7 µm, and −83.9 ± 179.3 µm, respectively - Improvement of 2 or more steps in DRSS score was observed in 37.0% and 37.1% in IVA regimens vs. 15.6% in the laser group 148-week results: VIVID: - The mean change in BCVA in IVA arms was +10.3 and +11.7 compared to +1.6 L in the laser arm - 41.2% and 42.2% of eyes gained 15 or more L in IVA regimens vs. 18.9% in the laser group - CST changed −215.2 µm and −202.8 µm in the laser group vs. −122.8 µm in patients following the laser treatment - Improvement in DRSS score of more steps was observed in 44.3% and 47.8% of patients following IVA vs.17.4% of individuals in the laser group VISTA: - The mean change in BCVA in IVA regimens was +10.4 and +10.5 vs. +1.4 L in the laser group - 42.9% and 35.8% of participants treated with IVA gained 15 or more L compared to 13.6% treated by the laser - CST changed −200.4 µm and −190.1 µm in IVA arms vs. −109.8 µm in the laser group - Improvement in DRSS score of more steps was observed in 29.9% and 34.4% of patients following IVA vs. 20.1% of individuals in the laser arm During the VISTA and VIVID studies, the incidence of ocular and non-ocular AEs and SAEs were similar between examined groups.	[24,35]

Abbreviations: AEs, adverse events; BCVA, best-corrected visual acuity; CRT, central retinal thickness; CST, central subfield thickness; DME, diabetic macular edema; DRSS, diabetic retinopathy severity score; IVA, intravitreal aflibercept; L, liters; PRN, pro re nata (as needed); SAEs, serious adverse events. ^a^ BCVA was measured according to the ETDRS protocol. ^b^ The results for each group are provided in the order described in the “Study Design” Section.

**Table 4:** Summary of clinical trials assessing the use of faricimab in the treatment of nAMD.

Trial Name	Phase	Number of Participants	Study Design	Primary and Secondary End Measurements	Explanatory Comment ^a,b^	Reference
-	I	24 nAMD patients	Single IVF injection at a dose: (1) 0.5 mg (2) 1.5 mg (3) 3 mg (4) 6 mg Multiple-dose phase: (1) 3 injections of 3.0 mg IVF (2) 3 injections of 6.0 IVF 4-week follow-up	- Safety and tolerability of IVF - Change in BCVA and CST at 4 weeks	- The mean BCVA changed +7 and +7.5 L in single-dose groups and multiple-dose groups, respectively - The mean CST decreased −42 µm in participants treated with a single IVF dose compared to −117 µm in the multiple-dose group Faricimab was well-tolerated, and there were no AE related to the drug action.	[85]
STAIRWAY	II	76 nAMD patients	Dosing regimens: (1) 0.5 mg ranibizumab every 4 weeks (2) 6.0 mg faricimab every 12 weeks after 4 monthly injections (3) 6.0 mg faricimab every 14 weeks after 4 monthly injections (with re-selection in IVF arms based on disease activity in 24 weeks)	Primary: - The mean change in BCVA at week 40 Secondary: -Change in BCVA measured every 4 weeks - Number of participants with +15, +10, +5, and 0 L gain vision - Mean decrease in CST, CNV lesion area, and leakage area	- In 40 weeks, mean BCVA change was +11.4 L in the IVR arm compared to +9.3 and +12.5 L w IVF arms, respectively - In 52 weeks, the mean BCVA gained +9.6 L after 12.9 injections, while in quarterly dosed faricimab, the mean BCVA changed +10.1 L after 6.7 injections, and in 4 monthly dosed faricimab, the arm mean BCVA gained +11.4 L after 6.2 injections - 33.3%/100% of participants gained/did not lose 15 or more L in the IVR group compared to 38.1%/95.2% and 39.3%/96.4% in INF regimens - The mean CST change was −126.3 µm in IVR arms compared to −138.6 µm and −121.3 µm in IVF groups - The mean CNV lesion size was reduced −4.6 mm^2^ in IVR, as well as −4.7 mm^2^ and −3.9 mm^2^ in IVF groups - Mean change in mean leakage lesion area was −5.3 mm^2^ in the IVR group vs. −5.0 mm^2^ and −4.3 mm^2^ in IVF arms Incidence of AEs and SAEs was similar between examined regimens across the study.	[73]
AVENUE	II	263 nAMD patients	Dosing regimens: (1) 0.5 mg IVR every 4 weeks (2) 1.5 mg IVF every 4 weeks (3) 6.0 mg IVF every 4 weeks (4) 6.0 mg IVF every 4 weeks until week 12 and then every 8 weeks (5) 0.5 mg IVR every 4 weeks until week 8 and then 6.0 mg IVF every 4 weeks	Primary: - Mean BCVA change at week 36 and mean BCVA change for patients with the incomplete response from groups (1) and (5) from weeks 12 to 36 - Percentage of patients with 15 or more L gain - Percentage of patients with BCVA at least/worse than 20/40 - Change in mean CST - Change in CNV lesion area and leakage	36-week results: - The mean BCVA change was +8.5 L in IVR groups and +10.9, +5.9, and +6.3 L in (2), (3), and (4) groups, respectively - 31.3% of participants gained 15 or more L in IVR group compared to 37.5%, 27.0%, and 22.7% in (2), (3), and (4) groups, respectively - Proportions of patients with BCVA 20/40 or better/worse than 20/40 was: 50.0%/7.8%, 50.0%/7.5%, 40.5%/16.2%, and 43.2%/9.1% for (1), (2), (3), and (4) groups, respectively - The mean CST changed −161.3 µm in the IVR arm compared to −152.2 µm, −190.7 µm, and −151.9 in IVF groups - The mean CNV area changed −2.6 mm^2^ in IVR arm vs. −3.4 mm^2^, −2.1 mm^2^, and −3.1 mm^2^ in (2), (3), and (4) groups, respectively - The mean change in leakage area was −5.2 mm^2^ in the IVR arm vs. −4.6 mm^2^, −3.2 mm^2^, and −5.4 mm^2^ in (2), (3), and (4) groups, respectively 36-week results with incomplete response groups (results are provided for (1) and (5) groups), respectively: - The mean change in BCVA was +2.1 vs. +0.6 L - 5.7% and 0% of patients gained +15 or more L - 22.9%/14.3% and 16.2%/13.5% of patients had BCVA of 20/40 or better/or BCVA worse than 20/40 - The mean CST changed −13.0 µm vs. −36.5 µm - The mean CNV area changed −0.5 mm^2^ vs. −1.4 mm^2^ - The mean area of leakage decreased −1.9 mm^2^ vs. −2.0 mm^2^	[65]
TENAYA and LUCERNE	III	1329 nAMD patients	(1) 6.0 mg IVF up to 16 weeks after 4 initial doses in 4-week intervals, and then based on the assessment of disease activity at 20- and 24-week doses in 8-, 12- or 16-week intervals until week 60 (2) 2.0 mg IVA in 8-week intervals	Primary: - Mean change in BCVA at week 48 (non-inferiority margin of 4 L) Secondary: - Proportions of patients treated in 8-,12- and 16-week intervals - Percentage of patients gaining ≥ 15, ≥10, ≥5, or ≥0 L and patients with loss ≥ 15, ≥10, or ≥5 L; patients gaining 15 or more L or achieving BCVA ≥ 84 L - The percentage of patients with BCVA 20/40 equal or better, and BCVA 20/200 or worse. Change in CST - Change in CNV lesion and leakage areas - Change in NEI VFQ-25 score - Incidence and severity of AEs	48-week results: TENAYA: - The mean BCVA change was +5.8 L in IVF groups and +5.1 L in the IVA regimen - The mean CST change was −136.8 μm in IVF regimens and −129.4 μm in the IVA group - Proportion of IVF-treated patients with 8-week, 12-week, and 16-week injection intervals was 20.3%, 34.0%, and 45.7%, respectively - The mean change in CNV lesion and leakage areas were comparable between IVF and IVA groups LUCERNE: - The mean change in BCVA was +6.6 L in IVF groups and +6.6 L in the IVA arm - The mean CST change was −137.1 in IVF groups and −130.8 μm in the IVA arm - Proportion of IVF-treated patients with 8-week, 12-week, and 16-week injection intervals was 22.2%, 32.9%, and 44.9%, respectively - The mean change in CNV lesion and leakage areas were comparable between IVF and IVA groups Summary analysis: - 45.3% and 78.7% of participants received IVF in 16- and 12-week intervals, respectively - 20.0–20.2% of patients in IVF groups gained 15 or more L compared with 15.7–22.2% in the IVA arm - NEI VFQ-25 score was also comparable between the groups In both studies, there were no significant differences among AEs between the examined groups.	[16]

Abbreviations: AEs, adverse events; BCVA, best-corrected visual acuity; CNV, choroidal neovascularization; CRT, central retinal thickness; CST, central subfield thickness; DME, diabetic macular edema; IVA, intravitreal aflibercept; IVF, intravitreal faricimab; IVR, intravitreal ranibizumab; L, liters; NEI VFQ-25, national eye institute visual function questionnaire; SAEs, serious adverse events; ^a^ BCVA was measured according to the ETDRS protocol. ^b^ The results for each group are provided in the order described in the “Study Design” Section.

**Table 5:** Summary of clinical trials assessing the use of faricimab in the treatment of DME.

Trial Name	Phase	Number of Participants	Study Design	Primary and Secondary Outcomes	Explanatory Comment ^a,b^	Reference
BOULEVARD	II	229 diabetic patients with center involving DME (168 treatment-naive and 61 non-treatment-naive)	Dosing regimens in treatment-naive patients: (1) 6.0 mg IVF every 4 weeks up to week 20 (2) 1.5 mg IVF every 4 weeks up to week 20 (3) 0.3 mg IVR every 4 weeks up to week 20 with follow-up period up to week 36 Dosing regimens in non-treatment-naive patients: (1) 0.3 mg IVR every 4 weeks up to week 20 (2) 6.0 mg IVF up to week 20 With a follow-up period of up to 36 weeks	Primary: - Mean change in BCVA in treatment-naive patients at week 24 Secondary: - Proportion of patients with 15 or more BCVA L gain - Mean change in CST - Improvement in 2 or more steps in the DRSS score	Results regarding treatment-naive patients: - The mean BCVA changed +13.9 and + 11.7 in IVF groups compared to +10.3 L in the IVR arm, respectively - The mean CST changed −204.7 µm in the IVR group vs. −217.1 µm and −225.8 µm in IVF regimens - Improvement in at least 2 steps in the DRSS score was achieved in 12% of patients treated by IVR vs. 28% and 39% of participants in IVF groups - 35.3% of patients in the IVR arm gained +15 or more L vs. 36.0% and 42.5% of participants in IVF regimens Results regarding non-treatment-naive patients: - The mean BCVA gained +8.3 and +9.6 L in IVR and IVF groups, respectively - The mean CST changed −148.0 µm in IVR regimen vs. −186.6 µm in IVR group - The DRSS score was improved in at least 2 steps in 23% of participants in both groups - The number of individuals with CST of 325 µm or less at week 24 was 53.6% in the IVR arm and 87.0% in the IVF arm Ocular and systemic AEs and SAEs were similar among examined groups.	[15]
YOSEMITE and RHINE	III	1891 diabetic patients with center involving DME	Dosing regimens: (1) six-monthly injections of 6.0 mg IVF at a dose of 6.0 mg and then every 8 weeks (2) four monthly injections of 6.0 mg IVF and then with PTI schedule (intervals were regulated in the range of 4–16 weeks based on the disease activity (3) five monthly IVA injections at a dose of 2.0 mg and then every 8 weeks	Primary: - Mean change in BCVA over weeks 48, 52, and 56 (non-inferiority margin of 4 L) Secondary: - Percentage of patients in PTI groups receiving faricimab in 4-, 8-, 12-, and 16-week intervals - Percentage of participants with CST less than 325 µm and with the absence of IRF or SRF - Number of patients with 15, 10, 5, and 0 letter vision gain and 15, 10, and 5 L vision loss - Number of patients with BCVA 20/40 or better - Percentage of patients with 2 or more step improvement in DRSS at week 52 Safety and tolerability of IVF	YOSEMITE: - The mean BCVA change was +10.7 and +11.6 L in IVF groups compared to +10.9 L in the IVA arm - 10.8%, 15.4%, 21.0%, and 52.8% of patients in the PTI arm received IVF injections at 4-, 8-, 12-, and 16-week intervals - The mean CST changed −206.6 µm and −196.5 µm in the IVF groups compared to −170.3 µm in the IVA arm - In 77–87% and 80–82% of patients, CST less than 325 µm was observed compared to 64–71% in the IVA arm - IRF was absent in 42–48% and 34–43% of participants in IVF groups and 22–25% in the IVA arm - The percentage of patients with 2 or more step reduction in the DRSS score was 46.0% and 42.5% in IVF groups and 35.8% in the IVA group RHINE: - The mean BCVA change was +11.8 and +11.8 L in IVF regimens vs. + 10.3 in the IVA group - 13.3%, 15.6%, 20.1%, and 51.0% of patients in the PTI arm received IVF injections at 4-,8-,12-, and 16-week intervals - The mean CST was reduced −195.8 µm, −187.6 vs. −170.1 µm in the IVA group - In 85–90% and 83–87% of patients CST less than 325 µm was observed compared to 71–77% in the IVA arm - IRF was not detected in 39–43% and 33–41% of participants in IVF groups vs. 23–29% in the IVA arm - The percentage of patients with 2 or more step reduction in the DRSS score was 44.2% and 43.7% in IVF groups and 46.8% in the IVA group In both studies, the incidence of AEs and SAEs was similar between study groups.	[17]

Abbreviations: AEs, adverse events; BCVA, best-corrected visual acuity; CST, central subfield thickness; DME, diabetic macular edema; DRSS, diabetic retinopathy severity score scale; IRF, intraretinal fluid; IVA, intravitreal aflibercept; IVF, intravitreal faricimab, IVR; intravitreal ranibizumab; L, liters; SAEs, serious adverse events; SRF, subretinal fluid. ^a^ BCVA was measured according to the ETDRS protocol ^b^ The results for each group are provided in the order described in the “Study Design” Section.

## Data Availability

Not applicable.
